# The development of highly dense highly protected surfactant ionizable lipid RNA loaded nanoparticles

**DOI:** 10.3389/fimmu.2023.1129296

**Published:** 2023-02-27

**Authors:** Ramon González-Rioja, Vivian A. Salazar, Neus G. Bastús, Victor Puntes

**Affiliations:** ^1^ Institut Català de Nanociència i Nanotecnologia (ICN2), Consejo Superior de Investigaciones Científicas (CSIC), The Barcelona Institute of Science and Technology (BIST), Universitat Autònoma de Barcelona (UAB), Barcelona, Spain; ^2^ Centro de Investigación Biomédica en Red (CIBER) en Bioingeniería, Biomateriales y Nanomedicina, Centro de Investigación Biomédica en Red en Bioingeniería Biomateriales y Nanomedicina (CIBER-BBN), Madrid, Spain; ^3^ Malalties Infeccioses, Nanopartícules farmacocinétiques, Vall d’Hebron Institut de Recerca, Barcelona, Spain; ^4^ Institució Catalana de Recerca i Estudis Avançats (ICREA), Barcelona, Spain

**Keywords:** ionizable lipid nanoparticles, RNA-loading, drug delivery carriers, pharmacokinetics, biodistribution and clearance

## Abstract

The long quest for efficient drug administration has been looking for a universal carrier that can precisely transport traditional drugs, new genomic and proteic therapeutic agents. Today, researchers have found conditions to overcome the two main drug delivery dilemmas. On the one side, the versatility of the vehicle to efficiently load, protect and transport the drug and then release it at the target place. On the other hand, the questions related to the degree of PEGylation which are needed to avoid nanoparticle (NP) aggregation and opsonization while preventing cellular uptake. The development of different kinds of lipidic drug delivery vehicles and particles has resulted in the development of ionizable lipid nanoparticles (iLNPs), which can overcome most of the typical drug delivery problems. Proof of their success is the late approval and massive administration as the prophylactic vaccine for SARS-CoV-2. These ILNPs are built by electrostatic aggregation of surfactants, the therapeutic agent, and lipids that self-segregate from an aqueous solution, forming nanoparticles stabilized with lipid polymers, such as PEG. These vehicles overcome previous limitations such as low loading and high toxicity, likely thanks to low charge at the working pH and reduced size, and their entry into the cells *via* endocytosis rather than membrane perforation or fusion, always associated with higher toxicity. We herein revise their primary features, synthetic methods to prepare and characterize them, pharmacokinetic (administration, distribution, metabolization and excretion) aspects, and biodistribution and fate. Owing to their advantages, iLNPs are potential drug delivery systems to improve the management of various diseases and widely available for clinical use.

## Introduction

The modern concept of drug delivery probably started with German Nobel laureate Paul Erlich’s “magic bullet” in 1907, a bullet that cannot miss its target (1, 2) and became common with penicillin in the early 20^th^ century (3, 4). The initial idea was to kill prokaryotes leaving eukaryotes unharmed, thus reducing damage to the body associated with uncontrolled biodistribution. Soon it was also developed to improve dosing and, with it, therapeutic effects. More recently, the concept was actualized for chemotherapy due to its severe side effects, and thus, the firsts Drug Delivery Systems (DDS) were developed to improve the transport of antitumoral drugs such as doxorubicin (5). Before, excipients allowed the drug to solubilize and properly reach their target, but their capacity was limited to solubility issues, with poor capabilities in directing and protecting the drugs during the journey to the target. Similarly, if the development of DDS was initially intended for solubilizing common drugs, it rapidly opened the possibility of loading other substances, such as genetic material or proteins –antibodies, enzymes, etc.-. These substances cannot be administered in a free form since they are highly immunogenic and rapidly biodegraded. Therefore, the full development of DDS will not only optimize the pharmacology of current drugs but also dramatically expand the pharmacopoeia we have available for the cure, which will have a clear impact on population health.

It is important to note that therapeutic effectiveness strongly depends on pharmacokinetic aspects, on how drugs travel and interact through the body, reach their target, perform their intended effect, are modified (metabolized), and excreted. Up to now, pharmacokinetic principles were based on small drug properties, where balanced hydrophobicity and hydrophilicity allow it to reach all corners of the body (6) so that reaching the target was assured at the expense of undesirable side effects. For example, after cisplatin injection, a common chemotherapeutic agent, 60% goes to the kidney causing nephrotoxicity, 36% irreversibly binds to albumin losing its activity, and only a small fraction of the remaining 4% reaches the tumor cell’s DNA and performs its therapeutic action (7). In this scenario, protecting the drug and carrying it to the region of interest is a natural evolution of pharmacology.

Together with the quest for efficient drug administration, chemists and nanochemists have searched for a universal carrier that can accommodate many different substances. In addition, the carrier has to be safe, more than its loading. DDSs have to be so safe that repeated administrations across one person’s life should not be a problem. Also, for practical reasons, they have to be easy to produce and easy to store.

As expected, DDS have been developed and exploited to enhance the delivery of drugs in treating several diseases showing potential benefits in terms of pharmaceutical flexibility, selectivity, dose reduction and minimization of adverse effects (8). In these platforms, different drugs, ligands and biomolecules can be combined by absorption (9), loading (10), coordination bonding (11), and entrapment (12) to perform different tasks. While DDS popularization started in the 80s and developed as a full academic and technological discipline, nanotechnology irrupted in the 2000s offering unprecedented control of mater structured at the nanoscale, allowing for the rapid and widespread development of new, more precise and more functional DDS. Interestingly, the initial vehicles were called microspheres and then renamed nanocarriers, even if some nanocarriers were bigger than some microspheres.

Among DDS, polymeric nanoparticles (NPs) were the first to be employed, showing significant therapeutic benefits but accompanied by polymeric toxicity, high cost, and lack of feasibility for scaling up. Besides, liposomes have traditionally been the more developed and implemented DDS. Lipid-based NPs made of lipids and surfactants (amphipathic molecules) are simple and safe, but there is typically a low degree of loading and structural fragility. They have become an up-and-coming delivery platform for hydrophobic and hydrophilic substances or a combination of both (13, 14). These carriers can penetrate abnormal tissue, remain for a long time and release their cargo drugs, increasing drug efficacy. For example, *in-vitro* studies by Wang et al. (15, 16) have shown the successful target delivery of resveratrol, a poor aqueous solubility drug and curcumin hydrophobic polyphenol in breast cancer. Recently developed lipid-based formulations included micro and nanoemulsions, self-emulsifying formulations, liposomes, lipid NPs and lipid-drug conjugates. Among the extensive range of lipid formulations, the solid lipid nanoparticles (SLNs) (17–19), the nanostructured lipid carried (NLCs) (14, 20), and the lipid-based nucleic acid therapeutics (21, 22) have probably centered the majority of the attention due to their successful activities toward multiples disease models. Other alternatives have been protein aggregates for unsoluble chemotherapeutic agents such as paclitaxel in Abraxane^®^ (23) or made of biological molecules such as polylactic-co-glycolic acid (PGLA) NPs, which has been considered one of the first universal nanocarrier platforms (24, 25).

In this work, we refer to those NPs made of a mixture of lipids, ionizable surfactants, and nucleic acids, which spontaneously form NPs by the electrostatic and hydrophobic collapse in water. These lead to highly dense and highly protective NPs, which can be singularized as ionizable lipid nanoparticles, iLNPs. These NPs can overcome the main biological barriers to cell transfection, including protection from endonucleases, and RNases, and selective targeting, when targeting moieties such as antibodies or aptamers are included, to improve the contact with the targeted tissues or cells (26), cell internalization, and intracellular release. Herein we focus on RNA-ionizable lipid NPs. RNA is not only a major player in genetic medicine that needs to be transported, but it is also a model of macromolecule that has to be protected until delivered inside the cells.

From a historical perspective, ionizable lipids evolved from years of working with permanently charged cationic lipids for transfection. The mechanism by which these cationic lipids capture nucleic acids is through complexing them by ionic interaction between the negatively charged phosphate groups on the nucleic acid molecules and a positively charged group on the lipid head, forming nucleotides-lipid complexes-, probably starting in 1987 with DOTMA, the first bi-layer forming cationic lipid, specifically designed and used for DNA transfection (27). However, due to its net positive charge, it presented unacceptable levels of toxicity at the doses necessary to produce therapeutically relevant levels of transgene expression, especially *in vivo* animal models, making its transition into clinical praxis impossible (28–30). In addition, the positive net charge induces plasma protein adsorption and rapid clearance by the immune system, negatively affecting transfection efficiency (31). They commonly present hemodynamic toxicities, such as the activation of the complement system and an increase in blood coagulation time (32–34). Ionizable lipids entered the field as an answer to this problem. These lipids are a pivotal element of the iLNP systems (35) and are characterized by having an ionizable functional group in their polar head with an acid-dissociation constant (pKa) below 7.0 (36). Their pH-tunable charge allows them to be neutral at physiological pH, minimizing their cationic burden and toxicity but be protonated at a lower pH at the maturing, acidified endosome. Their cationic nature inside the endosome will help its break and escape by enabling the interaction with the anionic membrane lipids (and subsequent formation of non-by-layer phases), allowing cytosolic delivery. Indeed, the ability to activate and deactivate the ionizable lipid cationicity, when necessary, enables it to adapt to the needs of the synthesis (charge on), distribution within the body (charge off) and escape from the endosome (charge on). These positively charged vesicles present the advantages of a liposome-mediated transfection (e.g., fusion with the cell membrane, protection from degradation, digestion, opsonization, etc.) and of a cationic-mediated transfection (e.g., complex formation with nucleic acids, association with the negatively charged cell surface).

Today, iLNPs have proven to be an efficient vehicle for effectively delivering RNA inside the cells, opening the doors to gene therapy. Their rapid implantation in several medicines already approved for human use is an unprecedented success within the community of nanomedicine, drug delivery and gene therapy. Though, to date, only three different systems of RNA delivery are approved by the FDA: antitumoral Patisiran and two RNA COVID-19 vaccines (Pfizer/BionTech and Moderna) (37), and many others are under clinical trial. Encouraged by the successful application of the SarCov2 mRNA-lipid vaccines produced by Moderna and Pfizer companies, the high biocompatibility of the lipid nanocarriers is being explored to treat many other diseases. Thus, in a continuous effort to cure other viral diseases, other mRNA vaccines are developing to fight against etiological agents such as Cytomegalovirus, Syncytial respiratory, or influenza viruses (Trial number: NCT05085366, NCT05127434, NCT04956575). In the field of cancer disease, the pharmaceutical companies Moderna and BioNTech are advancing in the commercialization of a potent therapy based on mRNA vaccines for melanoma; their clinical trials are in phases 1 and 2, respectively (Trial number: NCT0389788, NCT04526899). The multifunctional characteristics of the iLNPs also have been advancing in treating solid tumors. In a lack of successful results compared to the CART cell therapy in liquid tumors, the BioNTech company has developed an RNA-based CAR-T cell therapy to counter the accelerated growth in the Gastric, Pancreatic, Ovarian, and Biliary Tract Tumors (Trial number: NCT04503278) (38).

## Ionizable lipid nanoparticles: Structure, composition and characterization

Ionizable Lipid NPs consist of nanometrically sized particles (39) could be understood as a dense condensation of surfactants, lipids and genetic material. These NPs can be synthesized quickly and efficiently to encapsulate genetic material with high efficiency (high density) and have sufficient stability and robustness to travel through the body to their destination without being degraded or opsonized, protecting their cargo, and able to carry out an efficient cytoplasmic delivery of the genetic material. Their standard composition consists of 5 different compounds: i) the genetic material which has to be delivered, ii) an ionizable cationic lipid to interact with the genetic material and render it hydrophobic, iii) a helper amphipathic molecule, usually a phospholipid such as DSPC, iv) a helper sterol lipid, in the majority of cases Cholesterol, to making the structure more robust, and v) a PEG-lipid at the particle surface for surface stabilization and to avoid NP aggregation and opsonization. The ratio between these components varies among different formulations, but typically it can be around: ionizable lipid 50%mol (60%mass), phospholipid 10%mol (15%mass), Cholesterol 38%mol (15%mass), PEG-lipid 1,5%mol (8%mass).

These components interact and spontaneously structure themselves through a self-assembly process based on the ethanol injection method, which consists of rapid mixing of an ethanol phase, where the lipids are dissolved, into an aqueous phase, where the nucleic acids are dissolved in an acidic buffer. This rapid mixing induces the sudden supersaturation of the lipidic molecules, which leads to burst nucleation and their assembly into NPs, trapping the surfactants and genetic material (36). The process by which the genetic material is encapsulated in the lipid structure takes place in the first steps of the synthesis process when the ethanolic phase is mixed with the aqueous phase and self-assembly of lipids occurs. The first force that drives this self-assembly is an electrostatic interaction between the polar head of the positively-charged ionizable lipids at acidic pH and the negative charges of the nucleic acid chains at the working pH (typically around 4), and the second is the increase in polarity of the lipidic solvent by the addition of water, expulsing lipidic material from the liquid phase into the NPs. Thus, as the polarity of the solvent progressively increases, inverted micelle-like structures coalesce, interacting with the rest of the lipids and surfactants, which at the NP surface closes the particle in a spherical form making the NPs soluble in water.

**Figure 1:**
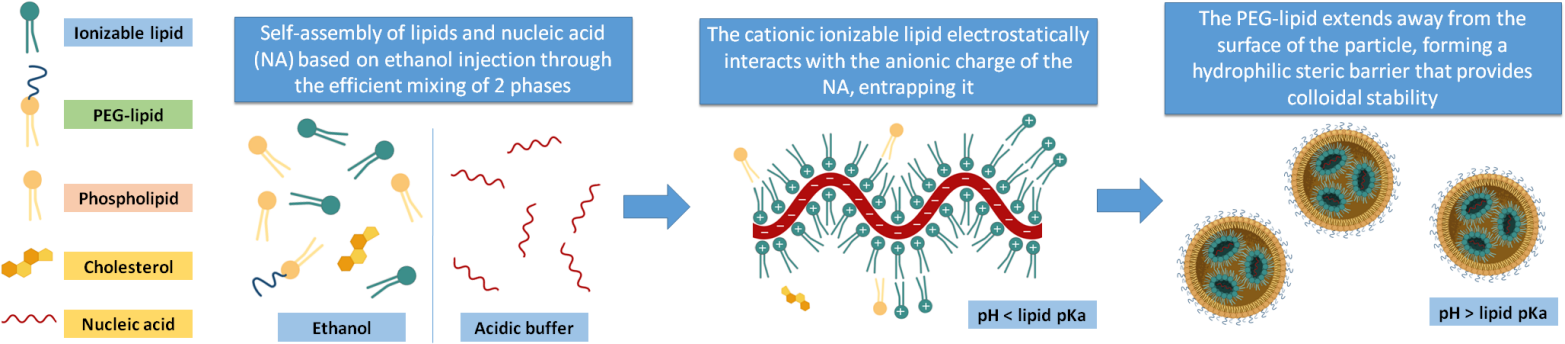
Main components of the iLNPs. The lipid components are dissolved in an ethanolic phase, and the nucleic acid is in an acidic buffer. When these two phases are efficiently mixed, the ionizable lipid gets protonated and electrostatically interacts with the anionic charges of the nucleic acids while the rest of the lipids self-assemble to form the iLNP structure. When the formation is complete, the pH of the batch can be brought to a value higher than the ionizable lipid pKa.

The PEG-lipid anchors to the NP surface’s lipidic domains while extending its water-soluble part away from the NP, forming a hydrophilic steric barrier that provides colloidal stability and prevents NP aggregation and opsonization (40). The whole phase mixing and complexation of the nucleic acid is done at pH<<pKa of the ionizable lipid so that it is cationic-charged nature, and the entrapment efficiency of the nucleic acid is maximized. Afterwards, once the synthesis is complete, the pH can be adjusted to physiological value since the nucleic acid is already complexed and integrated inside the NP. In this way, a neutral charge of the vehicle is achieved and significantly minimizes the cationic burden and its related toxicity that the particles would experiment with once they are administered (**Figure 1**).

An essential part of this process is how efficient and fast the mixing between these two phases is, since it determines size, and size plays a decisive role in NP biodistribution, delivery efficiency, and transfection potency (41). As shown by Belliveau et al.in 2012 (42) when investigating the influence of the flow rate on the iLNP particle size, the NP size decreased as the flow rate of the injection of the ethanolic phase into the aqueous phase increased. Indeed, there is an universal tendency for NPs to decrease surface energy (surface-to-volume ratio) by growth. Because of that, the PEG-lipid is employed to reduce the surface energy and stabilize the NPs. Multiple studies have shown that increasing the molar ratio of the PEG-lipid, by stabilizing NPs against aggregation yields significantly smaller iLNPs, independent of other lipid components (41–43). This indicates that without PEG, or other similar biocompatible polymers, the NPs would continuously aggregate and grow until complete phase separation.

Besides size, as discussed in the previous section, a determining functional parameter is the charge, a fundamental aspect of iLNPs, which should be positively charged during iLNPs formation to allow nucleic acid complexation, neutral at physiological pH for its administration, and positively charged at the acidified, maturing endosome for membrane disruption. The pKa value of the ionizable lipid will be the factor that determines the charge on the iLNP under the different pH conditions, which has to find a balance between (44, 45) i) being acidic during RNA trapping, ii) being neutral at physiological pH, to minimize toxicity and avoid rapid immune-clearance, iii) being as positively charged as possible at late endosome stage to maximize the interaction with the endosome’s membrane and its disruption. Also important to consider the effect produced by absorbing protons during endosome acidification, inducing proton sponge effects.

**Figure 2:**
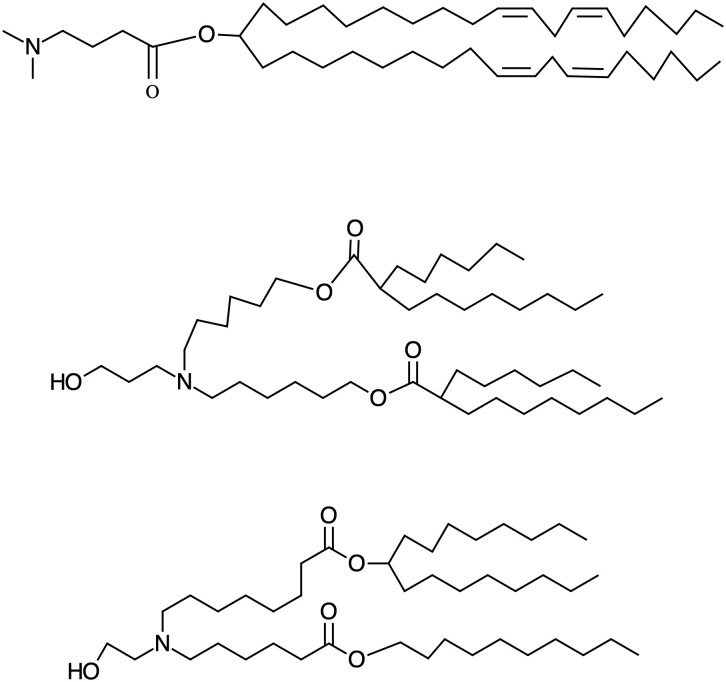
Structure of the approved Ionizable lipids. From up to down DLin-MC3-DMA, Alc-0315 and SM-102, the ionizable lipids present in Onpattro, Comirnaty (BioNTech/Pifizer) and Spikevax (Moderna), respectively, with the pKas: 6.44, 6.09, 6.75, respectively. The number of hydrophobic tails has geometrical consequences in the structure of the iLNPs contributing to the determination of size and robustness.

The pKa value in which this balance is optimal is not a universal value for all lipids and depends on the iLNPs formulation and the nucleic acid sequences they carry. However, several studies demonstrated that a pKas between 5,5 and 6,5 tend to show maximal potency *in vivo (* 21, 46–48). Regarding their chemical structure, as a rule of thumb, one can say that small head groups of the ionizable lipid, such as dimethylamino-based, show higher transfection efficiencies compared to higher substituted moieties, which increase their steric hindrance as well as affecting the pKa (49–51). So it is the case of DLin-MC3-DMA, the ionizable lipid part of the patisiran (Onpattro^®^) formulation. It is important in the history of the development of ionizable lipids and corresponding NPs since, when first synthesized, it exhibited an improvement in the potency of more than two orders of magnitude compared to the previous benchmark formulation (DLinDMA), which allowed the TTR02 (later known as patisiran) formulation to transition into clinical development (45). Notably, the structure and formulation of DLin-MC3-DMA laid the groundwork for further iLNPs development. Currently, the search for new biodegradable, ionizable lipids with more potency or different properties is at the center of research to advance iLNPs, and this effort is yielding a large array of diverse and exciting types of iLNPs to adapt them to organs and diseases (52). The structures and pKas of the ionizable lipids present in the approved iLNP formulations appear in **Figure 2**.

They share branched (bulky) lipophilic moieties and hydrophilic amino terminations. This geometry allows for a cone-like conformation of the amphipathic molecules and of the cationic-anionic lipid pair that occurs once the ionizable lipid interacts with the lipids of the endosomal membrane. This non-by-layer, cone-like conformation of the amphipathic molecules favors high surface curvature and is responsible for iLNP disruption. Once the endosome and the iLNP have been disrupted, the pH goes back to 7, and the iLNP release its cargo so that free mRNA can enter ribosomes for expression. This disintegration is probably simultaneous with the endosomal disruption and mediated by the many (negatively) charged and detergent-like (amphipathic) compounds inside the cell –indeed, all proteins have hydrophilic and hydrophobic domains- interfering with the amphipathic molecules of the iLNPs.

**Figure 3:**
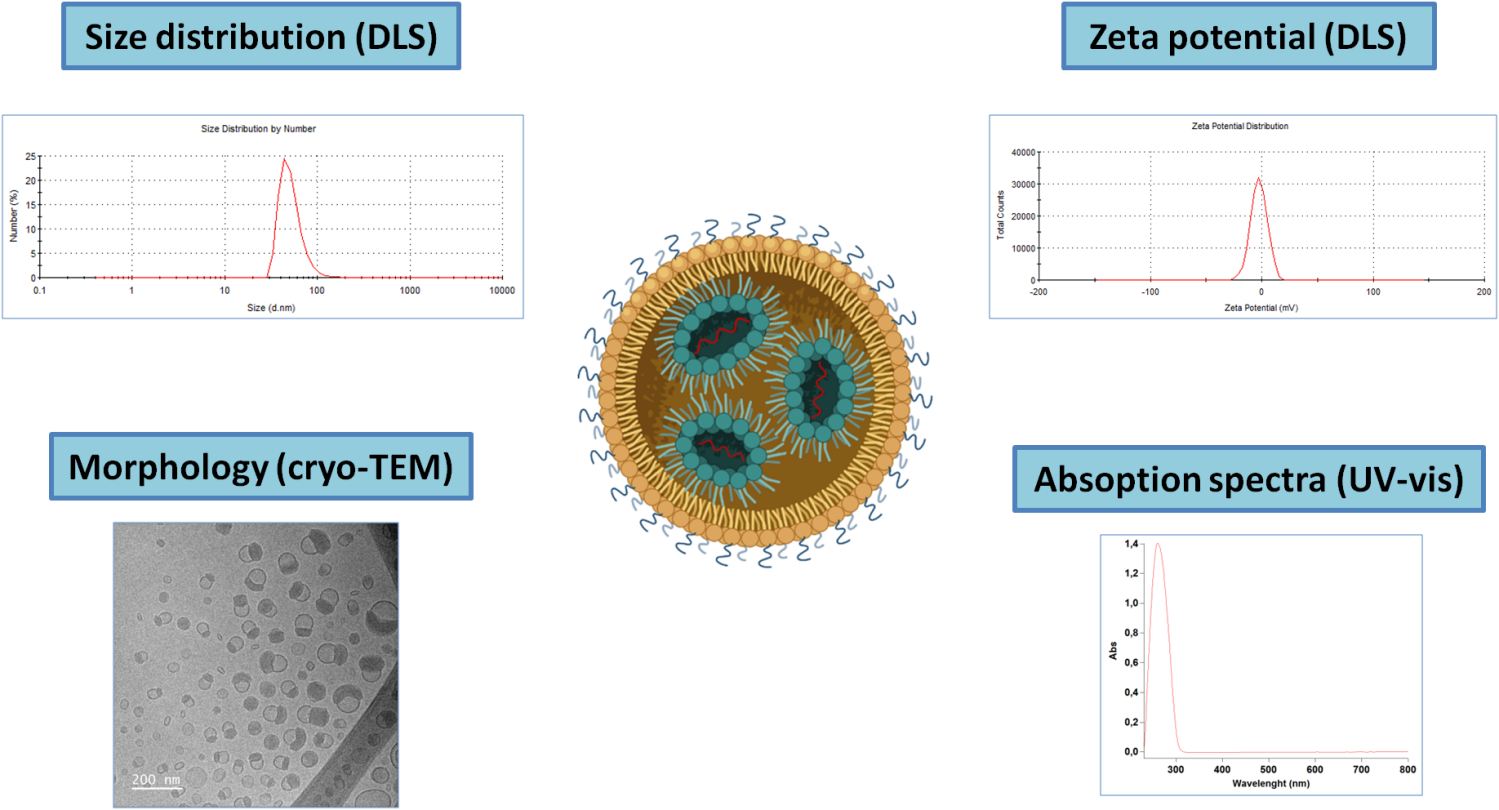
Schematic representation of the main techniques for iLNPs characterization. Note that for the UV-vis absorption spectra, the signal corresponding to the nucleic acids present in the sample will account for the majority part of the signal.

Due to their reduced size and complex and unstable (dynamic) nature, their characterization can be a serious challenge, especially in the biological matrix (**Figure 3**). The standard parameters to evaluate include particle size, surface charge (ζ-potential), drug content and surface state (composition and conformation). Particle size, polydispersity index and charge analysis can be measured by dynamic light scattering (DLS) and associated ζ-potential with the main advantage of not being time-consuming. Besides, electron microscopy allows for high-resolution observation of these NPs. However, they are frail in high vacuum and under the electron beam, and therefore cryo-TEM is often employed, providing 2D images of stable frozen-hydrated particles. Alternatively, low electron beam energy and staining also allow observation of the iLNPs morphology (53). These iLNPs can also be fluorescently-labelled for their visualization and quantification in fluorescence microscopy and spectroscopy techniques (54). Concentration and composition are also studied using thermogravimetry and differential scanning calorimetric analysis (55).

Information about the internal structure of the systems can be obtained through X-ray (SAXS) and neutron (SANS) small angle scattering (55). In the past decade, SAXS has been shown to be very useful at providing information about the fine structure in self-assembled soft matter materials, like iLNPs (56–59). Still, the full understanding of the nanoscale organization of the lipids and genetic material in the interior of the particle remains yet to be achieved (60). It has been observed that depending on operational factors or compositional changes, the synthesis yields different arrangements of iLNPs, such as multilamellar structures (61), Ia3d and Pm3n cubic phases (62), and other types of non-lamellar structures where the RNA molecules are inside aqueous cylinders (63).

## Ionizable lipid nanoparticles: Pharmacokinetics

It is well accepted that the potential use of iLNPs in medicine is determined by the pharmacokinetic (administration, distribution, metabolization and excretion) aspects that govern iLNP behavior, which is different from previous drugs. Pharmacokinetics describes what the body does to the drug rather than what the drug does to the body; the latter would be pharmacodynamics. The field of pharmacokinetics has developed with the implantation of small-molecule drugs as principal therapeutic agents. This is because small molecule drugs can distribute across the body and enter inside the cells. However, everything changes when we pretend to employ large and structured substances such as proteins, genetic material or nanoparticles. Because of that, new pharmacokinetic models, which describe the behavior of these materials once injected until they are excreted, have to be developed for the proper implementation of new medical substances and materials into the clinic practice, taking into account that the biochemical composition of NPs and its entrance route into the human body, determines the final activity of these NPs (64). We focus on the existing clinical trials and *in-vivo* experimental models using RNA-lipid carrier systems. Many DDS enter the body *via* inhalation, oral ingestion, topical (cutaneous and ocular) application, and parenteral administration.

Herein, we first analyze the different transformations iLNPs may suffer, such as aggregation, interactions with proteins and disintegration/dissolution, and then comment on their biodistribution and excretion since the latter strongly depends on the formers. These alterations significantly impact their behavior and must be considered for their intended use in medicine. Therefore, research on iLNPs effects should strive to correlate with how they interact, evolve and are transformed during their exposure to the human body. That is, during their Administration, Distribution, Metabolization and Excretion (ADME) phases.

### Transformations and metabolization

Regarding nanosized objects in general, due to interactions between NPs and components from the biological medium, NPs are known to suffer different alterations when applied. Indeed, NPs are intrinsically out of equilibrium, and transformations such as Ostwald ripening, NPs collapse, and over-grow always tend to occur. When administered, the NP’s environment radically changes from low electrolytic NP concentrated media to a media full of cells, proteins and electrolytes. The main alteration they may suffer is the loose of colloidal stability and consequent aggregation. This loss of colloidal stability and subsequent aggregation and expulsion from the media has a dramatic effect on the abilities of NPs to travel through the body and to be well dispersed in organs and tissues, provoking, in too many cases, the lack of -or unexpected- biological results (65, 66). Note that the final size of NP, which will reach the cells, is also determined by the interaction with the biological matrix, ultimately determining the distribution and kinetics of delivery.

Several factors cause the aggregation of colloidal NPs; for instance, the initial concentration of NPs, their chemical nature and the ionic strength of the medium (67). The most widely employed strategy to passivate the surface of nanosized objects, in general, has been modifying the NP surface with hydrophilic polymers as polyethyleneglycol (PEG), which acts as a steric barrier that minimizes interactions at the NP surface, indispensable to stabilize the surface and allow NPs to exist isolated in solution. This steric barrier “closes” the surface of the NP, provides colloidal stability and facilitates the small and narrow size distribution. However, the PEGylation of the NP surface dramatically difficult the interaction with cellular membranes, reducing the necessary close contact required for endocytosis. This double effect has been called the “PEG-dilemma” (68–71). Although alternative strategies are proposed, like cleavage of a PEG moiety (72), this problem is majorly addressed by a strategy based on “reversible PEGylation”, where a lipid-containing PEG slowly detaches from the NP surface once administered. This allows to take advantage of the disposing of the high PEG concentration needed for small and monodisperse synthesis and distribution, and the lower PEG concentration needed to have a good cellular uptake at the moment when the NP reaches the target organ. Note that an increase in the concentration of PEG also influences its conformation and protective effects, increasing the circulation time of the iLNPs (71). Indeed, a completely PEG-covered iLNP surface will dramatically inhibit the interaction with cells and serum proteins, modifying circulation time and biodistribution (68, 71). Such PEG-lipids remain integrated into the LNP structure during formation and under storage conditions, but in the presence of a lipid sink like in plasma, these PEG-lipids are stripped off the particle and into de medium, leaving the surface of the iLNP gradually unshielded (45). These PEG-lipids have short alkyl chains -which act as “hydrophobic anchors”-allowing a reasonable desorption rate once they enter blood circulation (71). The proper balance between a fully protected surface for iLNP synthesis and distribution, and a relatively unshielded surface for interactions with cell membranes, is achieved by finding a compromise between alkyl chain length and PEG-lipid surface concentration.

Special mention deserves the interaction of NPs with proteins, adjusted by the size and concentration of PEG at the NP surface. When NPs are administrated into the body, they first interact with biological fluids. Depending on the administration site, the biomolecules that will interact with the NPs can vary: from lung surfactants when inhaled to the interstitial fluid when locally injected into blood plasma following intravenous administration (73). Proteins, which are the most important of these biomolecules, will adsorb -to some extent- on the NP surface, especially as PEG is removed, coating the surface with a new layer that will define the biological identity of the NP, the so-called “biomolecular corona”, or “protein corona” (74, 75) which can dramatically alter the surface properties of nanosized particles and determine their *in vivo* fate (76). Already in 2004, it was reported that the presence of proteins in physiological media affects the entry and intracellular localization of NPs in cells, thus modulating their potential biological effects and toxicity. Later on, the formation of a protein corona on top of the NP surface was observed to control biodistribution, uptake and biological response, transforming them from innocuous to toxic or vice versa. This surface coating can be formed by hundreds of biomolecules, including albumin, apolipoproteins, immunoglobulins, coagulation factors, and many others (77). Some of these biomolecules might associate almost irreversibly with the iLNP surface, in either their native or denatured form, affecting, de facto, all subsequent interactions. It has been proposed that the corona is comprised of both these tightly bound proteins (“hard” corona), which presumably bind directly to the iLNP surface with high affinity, and also a looser, more dynamic layer (“soft” corona) which constantly exchanges with proteins in the environment (78). The hard and soft corona are both considered relevant in determining iLNP interactions with cells (73).

Traditionally, for biomedical applications, this corona has only been conceived as a disruptive effect that hinders the functionality of nanoparticles, provoking underestimated side effects like loss of colloidal stability, aggregation, sedimentation or rapid clearance by the immune system. But the protein corona also plays an active role in deciding the destination organ for delivery and accumulation of the particles. For iLNPs, it has been shown that in Onpattro, there is a close relationship between the protein corona and the target organ of delivery, mediated through the adsorption of Alipoprotein E onto the iLNP surface. Authors proposed that binding to ApoE will act as a highly effective targeting ligand by binding to lipoprotein receptors on the surface of hepatocytes, triggering the uptake by hepatocytes (45, 79). This relationship between the protein corona and the biodistribution of NPs could allow the fate of the particles to be actively altered. Indeed, the multifunctional physicochemical properties of lipids can be designed to target different body tissues. Min Qiu et al. (80) have achieved a lung-selective delivery in mice with the use of a series of ionizable lipids containing an amide bond in the tail which changes the interactions between plasma proteins in contrast with other types of lipids, like the ones with an ester bond in the PEG lipidic tail (as those present in the approved formulations), which easily accumulate in the liver (81). These are exciting and promising results for improving the delivery of iLNP beyond these organs, and other relationships between lipid composition and biodistribution should be carried out in the future. Active targeting by grafting a specific moiety, that is, the ligand of an over-expressed receptor onto the NP surface, is a very appealing strategy that, to this day, fails to impact the biodistribution drastically. However, once the NPs have reached the organs, their uptake can be influenced by targeting moieties (75)

### Degradation and disintegration of iLNPs

It is well-known that NPs can dissolve in certain dispersing media (82–84). The extent of their dissolution depends not only on their intrinsic properties, such as size and shape, but also on characteristics of the surrounding media, including pH and ionic strength, as well as the presence of organic matter (85, 86). Thus, while small NPs can be preserved in solution in the appropriate (as–synthesized) conditions for a long time, they may also be prone to rapid degradation in physiological media. This is why iLNPs can be kept for a long time in storage conditions while in hours disintegrates once in the body. Indeed, it has been reported that it is below c.a. 30 nm in diameter (87), where NPs cannot support the high surface energy anymore and tends to dissolve. The driving force behind dissolution strongly depends on the solubility of the constituent ions in a given environment and their concentration gradients in the solution. This phenomenon, enhanced at the nanoscale, is referred to as the Gibbs-Thomson effect, and in NPs manifests as Ostwald ripening, where NPs in solution spontaneously dissolve or grow due to concentration gradients, becoming progressively larger and more polydisperse. Controlled release of matter from an NP is illustrated in the Noyes-Whitney equation, which relates the rate of dissolution to the properties of the components and the dissolution medium. If the released components are removed from the equilibrium because, for example, are used in competitive reactions or simple diluted in the body, the system is moved away from the saturation point, reaches sink conditions, and the NP tends towards complete dissolution. For a given mass, the kinetics of dissolution will be proportional to the specific surface area and the coordination of the constituents at that surface (which decreases with size). NPs have to release their cargo at the appropriate rate and quantity: larger NPs may release them too slowly and too much, while the smaller ones may release them too fast and an insufficient amount of it. Thus, the reactivity of the NP has to be adjusted to persist more or less inside the different parts of the body.

### Biodistribution and fate of iLNPs

The primary purpose of using these iLNPs is to cross natural barriers, interact with the target cell, and deliver the treatment efficiently. The first natural barrier the iLNPs need to cross is the biological fluids, blood or lymph, sweat or tear, and the corresponding extracellular matrix, consisting of macromolecules and minerals that change in different tissues, compartments, and health status (88). To overcome these barriers, some iLNPs can interact with particular matrix components that facilitate the interaction with the target cell and the entrance by endocytosis (87). The second natural barrier is the mononuclear phagocyte system, capable of recognizing foreign substances and commensal organisms when entering the body, labelling them with opsonins and enhancing uptake by phagocytic cells, such as kupffer cells in the liver. Basically, if NPs are recognized as a foreign substance, the innate immune response reduces their plasma half-life, decreasing the drug delivery efficiency to the target cell. However, the size range of NPs is that of recognition of the immune system which is supramolecular structures and molecular patters of few tens of nm, mainly found is viruses and bacteria, in such a way that the immune response to NPs can be complex from tolerance, to pro-inflammation to immunomodulation (89). As mentioned above, the lipid nanoparticle-surface functionalization with PEG minimizes opsonization and therefore the immune response and increases blood circulation time (90). Nonetheless, the activation of the immune system has been found against some of the iLNPs components as PEG. It has been reported that some patients can develop anti-PEG antibodies after a first dose of a PEGylated drug (anti-PEG immunoglobulin M (IgM)) (91), leading to rapid NPs clearance in the liver and spleen, removing the drug from circulation (92). Fortunately, PEG immunogenicity is not so prevalent and not so aggressive, while the rest of the approved iLNPs components are safe.

The precise behavior of these materials during their full-life cycle inside the body is still relatively unknown, with controversy about disparities between the *in vitro* and *in vivo* results. Additionally, subtle modifications of their nature -composition, size, shape and surface state- may have or not have a strong influence on their behavior, affecting their interaction with proteins (93), aggregation state (94), chemical transformation and degradation (95), and consequently biological responses (96). Once they are stable and do not aggregate when administered, their behavior is very different from small molecules, and they are subordinated to the many-body barriers to protect integrity. In addition to immunogenicity, which is somewhat tolerant to small molecules, the body is full of physical barriers. Considering intravenous administration, it is essential to note that the main blood vessels and capillaries in the body have a continuous lining of endothelial cells with pores of 6 nm. Besides, the fenestrated capillaries found in the intestine and some endocrine and exocrine glands may have pores up to 50–60 nm, while discontinuous capillaries, as those found in the liver, spleen and the bone marrow, have pores between 100–1000 nm, which is where typically NPs are found (97). Special attention deserves the tight junctions, including the blood-brain barrier, placenta and testis barrier, where pores smaller than 1 nm have been reported, where the hydrophobic nature of LNPs seems to favor translocation (98). In such conditions, small molecules can diffuse in-and-out from the blood vessels into the lymph, while the passive transport of large objects, like proteins and NPs, through these porous is negligible, and they tend to accumulate in organs of the mononuclear phagocytic system, such as the liver and spleen, which are the two usual places of NPs fate and accumulation (97, 99).

It is worth noting here that blood vessel and tissue permeability is altered during the course of diseases, allowing for the passive accumulation of NPs in those areas. Indeed, this passive accumulation can increase one order of magnitude the concentration of the drug in tissue (11). For example, in solid tumors, their rapid growth results in leaky vessels with large pores resulting from a defective angiogenic process, which facilitates NP accumulation in the absence of a functional lymphatic drain. This phenomenon, known as the Enhanced Permeability and Retention effect (100), is widely reported in the literature and has been exploited to accumulate nanocarriers in tumors (101) passively. Note that protein aggregates and cell debris are naturally found in solid tumors due to the EPR effect. Thus, by increasing NP circulation times, this passive accumulation has been employed to deliver therapeutic doses of drugs. It is the case of Doxil (liposomal doxorubicin), where the inclusion of PEG-1,2-distearoyl-sn-glycero-3-phosphorylethanolamine (PEG-DSPE) extended circulation time over 4- to a 16-fold enhancement of drug level (101). With this increased circulation time, the Doxil liposome formulation could accumulate more at the tumor site (101), achieving a more significant therapeutic effect (102, 103). Besides, blood and tissue porosity increases during inflammation, which allows NPs to accumulate in those sites (104).

Once the NPs reach their target cells, they must enter and deliver the cargo. The cytoplasmatic membrane is very robust and impermeable; things naturally enter either transported, typically ions and small molecules, or endocytosed, for proteins and larger objects. Endocytosis can be divided into pinocytosis (cell “drinking”) and phagocytosis (cell “eating”). Pinocytosis, commonly termed endocytosis, is when a fraction of the membrane is invaginated, and whatever is on its surface or around it is trapped. Endocytosis can be receptor-mediated or receptor-independent when the cell membrane recycles (105). Alternatively, substances like cationic detergents can permeate through the membrane, and large liposomes can fuse with the membrane. Both pathways often show toxicity, especially membrane permeation, being endocytosis the most benign way to introduce substances inside the cell. For the case of iLNP the successful endocytosis process is determined by their size, surface composition, and target cells (99). Once the iLNP reaches the target cell and is up-taken by endocytosis (45), the cargo must be released and reach the cytoplasm. It is important to note that the relationship between cellular uptake of NPs and transfection efficiency is not trivial since this is determined by the ability to escape from the endosome. Once engulfed into the cell by the endocytic process, the early endosomes mature into late endosomes and fuse with lysosomes decreasing pH and digesting its content for recycling (106). When the pH becomes smaller than the ionizable lipid pKa, it becomes positively charged again, enabling an electrostatic interaction between the iLNP and the negatively charged lipids of the cell membranes, as cationic lipids do.

**Figure 4:**
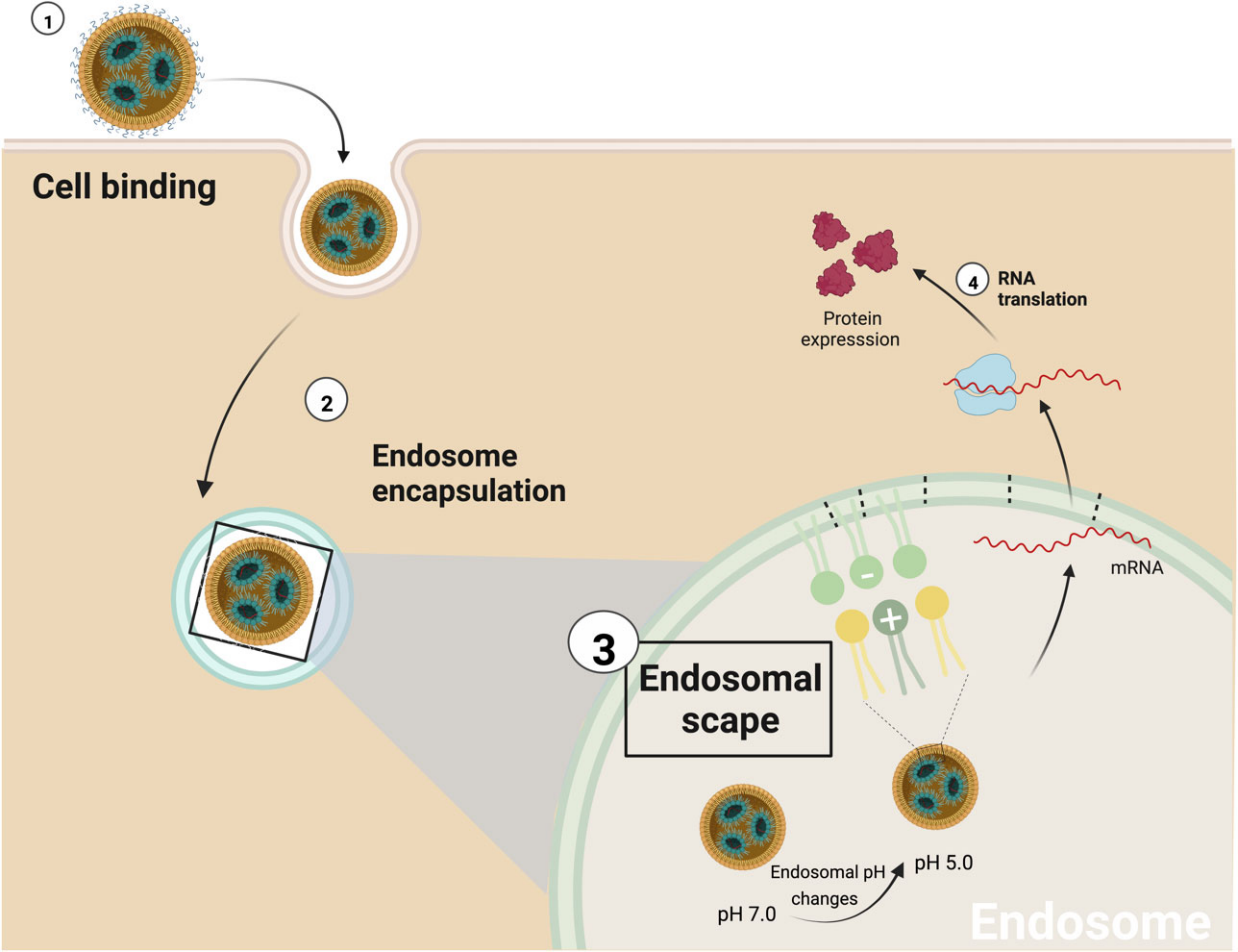
Schematic representation of endosomal scape mediated by iLNPs. 1) The first interaction between iLNPs and the target cell activates the endocytosis process, and 2) subsequently, the nanocarrier is encapsulated by the endosome. 3) As proton pumps reduce the pH of the endosome, the ionizable cationic lipid becomes progressively protonated (positively charged). As a result, it can interact with the endosome’s membrane anionic lipids to produce non-bilayer structures, disrupting the endosomal membrane and the iLNP structure and 4) releasing the mRNA into the cell cytoplasm.

This interaction can have disruptive effects, first on the NP structure and integrity, and second, on the endosome membrane, leading to its disruption (49, 107–109), promoting endosomal escape and cytoplasmatic release of the nucleic acid cargo (107). As the electrostatic interactions between ionizable lipid and RNA increase as pH decreases, it is difficult to imagine the liberation of the cationic surfactant associated with the RNA from the NP, indicating the presence of an excess of ionizable lipids in the iLNP formulation. It has been proposed that endosome disruption is achieved by forming non-bi-layer phases due to the electrostatic interaction between the lipids. Of particular interest is the HII inverted hexagonal structure since it’s been shown that this phase is not compatible with bilayers, and, as lipids tend to adopt it once they are mixed, membrane fusion and membrane disruption events are more likely to occur (35, 49, 107, 109). Regarding endosomal disruption, another possible factor playing a significant role in endosomal disruption would be the proton sponge effect. The proton sponge effect happens when during the maturation of endosomes, HCl influxes to decrease pH for digestion. The amine groups of the cationic lipid become protonated, capturing protons from the media that resist acidification. As a result, more protons are pumped into the endosomes, followed by passive entry of more chloride ions by osmosis, the consequent increase in ionic concentration, leading to osmotic water influx and swelling, and up to rupture of the endosomes -and endolysosomes-, releasing their contents into the cytosol (110). Likely, membrane disruption and proton sponge effects happen in parallel: while the excess of ionizable lipids perturbs the membrane, RNA-bonded ionizable lipids scavenge H^+^ intended for acidification. Once the cargo is delivered, pH returns to 7, quenching the cationic charge and leaving the genetic material ready for action. Finally, it is also important to note the clear difference that exists typically in cellular uptake between an *in vitro* system and an *in vivo* system, since *in vitro* conditions, the limitation of a low charge at physiological pH is much more relaxed, allowing the use of ionizable lipids with higher pKa, which can give a higher efficiency than *in vivo* (3). The endosomal escape mechanism mediated by the iLNPs, is illustrated in **Figure 4**.

### Excretion of iLNPs

Once the NP disintegrates, its components have to be processed and excreted from the body. DSPC and Cholesterol are both part of cell membranes; therefore, degradation and metabolism of these products will occur integrated within the natural processes of the cell. Nucleases metabolize the delivered genetic material to nucleotides of various lengths. In the case of the ionizable lipids, the three (DLin-MC3-DMA, SM-102 and ALC-0315) present in the approved formulations have ester bonds, which are degraded by hydrolysis, allowing their degradation into different metabolites that are more easily excreted or harmless. The introduction of ester bonds, stable at physiological pH, which are hydrolyzed by enzymes once inside the cell or tissues, is a widely used strategy to increase the biodegradability of these lipids, reducing their accumulation and possible consequent side effects (52). DLin-MC3-DMA is primarily metabolized by hydrolysis to 4-dimethylaminobutyric acid (DMBA) and excreted from the body through the urine (ONPATRO Assessment report EMA/554262/2018). On the other hand, PEG2000-C-DMG seems not to be extensively metabolized and is suggested to be eliminated unchanged through the hepatobiliary tract to the feces (ONPATRO Assessment report EMA/554262/2018). In the case of ALC-0159, the PEG-lipid present in Comirnaty, the BioNtech Pfizer vaccine, the primary route of metabolism appears to be related to amide bond hydrolysis, yielding N,Nditetradecylamine (COMIRNATY Assessment report EMA/707383/2020).

## Current challenges for ionizable lipid nanoparticles to be used in medicine

Regarding DDS, a common limitation is dosing. In systemic delivery, taking into account that there is a limitation of volume that can be injected in a single shot into the body (e.g. 10 mL/Kg for intravenous injection in mammals), the therapeutic dose might not be reached unless iLNPs have been previously concentrated because, during synthesis and storage, high concentrations lead to larger and polydisperse particles. Besides, *in vitro* simple tests, as those assessed in monolayer cell cultures, do not consider important factors such as organ vascularity, organ penetration and other differential properties given by the organ microenvironment (111), something that is not so critical for small molecule drugs but that is determinant for NP biodistribution and effects. Similarly, different *in vivo* models show variations between them, such as the different sizes of pores in vessels or different immunological responses, which may result in different efficacy for the same NPs depending on the model being used. Also, to be able to work with animals with implanted tumors, SCID (Severe Compromised Immunodeficiency) models are often used, which lack a fully functional immune system, significantly when its size ranges between virus and bacteria. In this context, 3D cell cultures have been proposed as suitable models to study the behavior of iLNPs in a particular environment due to the possibility of mimicking a controlled extracellular matrix and different organ regions (112).

Regarding the penetration and distribution inside organs, it is known that macromolecular carriers fail to penetrate deep into organs and tumors and are generally accumulated just some micrometers away from the vessels that transported them (113–115). Furthermore, solid tumor penetration is also challenging for small molecules (111). Finally, as carriers, they could carry the substance to the wrong place with high precision, producing unexpected side effects. Also, regarding safety, as the vehicle aims at universality, it will be applied repeatedly to the individual across their life, which may end up triggering pro-inflammatory immune responses towards the iLNPs or some of their components, for example, PEG (116, 117). Beyond parenteral application, in the following, we list some of the current iLNPs developments showing the universality of the vehicle’s ability to transport different sequences through different organs and portals of entry:

### Oral ingestion

The most preferred administration route for medical treatments is oral delivery. Several studies suggest that the lipid nanoparticle composition enchases their biodisponibility in the gastrointestinal mucosae following the natural entrance in the digestion process (118). Additionally, studies on the siRNA- lipid nanoparticle stability showed that the LPNs remained potent and stable after exposure to solutions with pH values as low as 1.2. However, future research needs to increase cargo delivery and improve the effectiveness once mucin is present in the intestines (119). Interestingly, new research conducted by Sung et al. (120), demonstrates the efficiency deliver of IL-22 mRNA -loaded iLPS administrated by oral route in a mouse model of acute colitis. The results showed a high level of expression of interleukin 22, the recovery of body weight, and an accelerated healing process in the colon tissues.

### Ocular applications

The main medicaments designed for ocular applications act in the anterior part of the eye. However, some degenerative processes, such as age-related macular degeneration, retinitis pigmentosa, and diabetic retinopathy, occur at the retina level in the posterior part of the eye. Here iLNPs provide sustained gene expression that could overcome these limitations (121). Recently, Wang and co-workers employed an iLNP to generate a cell-specific gene delivery system with sustained gene expression in the eye tissue (122).

### Cutaneous application

As an external barrier, skin homeostasis is a complex process. Therefore, a broad spectrum of topical medication treats diverse cutaneous diseases. Herein, iLNPs are a promissory agent to improve drug penetration through the stratum corneum. Although to date, no iLNP has been reported for commercial cutaneous applications, the efficient encapsulation of distinct cosmetic agents such as oils, vitamins, and antimycotic and anti-age compounds are the most common pharmaceutical approximations (123). As an approach to treating a chronic wound, Gainza and co-workers loaded the recombinant human epidermal growth factor (rhEGF) into an iLNP showing an essential recovery in healing grade and wound maturity (124).

## Conclusions and future perspectives

The iLNPs are a promissory concept to explore the efficient drug delivery of a broad range of substances, including genetic material, proteins and other NPs, decreasing collateral effects in healthy tissues and cells. However, despite the current knowledge on the subject being scattered and too heterogeneous, many recent discoveries and advances preclude the inevitable success of iLNPs. Indeed it has been predicted that Nanoparticulate DDS will be the most innovative and crucial cornerstones in pharmaceutical research, with a tremendous economic impact, where the possibility to adjust their physicochemical characteristics and increase the interaction with the target cell, and control the escape from the endosomal compartment allow for precision medicine. Thus, novel iLNPs will be developed with growing interest and improved pharmacokinetic profiles compared to standard drug delivery.

They are simple to produce, can load hydrophobic and hydrophilic substances, and are easily functionalizable with target moieties to ensure better precise delivery once an organ is reached. Indeed, encapsulating the genetic material into iLNPs is one of the fastest currently developing pharmaceutical technology. This success is the result of its clever design, which, in a biomimetic way, adapts to the different barriers and biological difficulties encountered along the way. All in all, in the near future, iLNPs may result in a quantum leap in medicine history.

## Author contributions

RG-R, VS, NB, and VP contributed to the design and implementation of the bibliographic research, the analysis and discussion of the literature and the writing of the manuscript. All authors contributed to the article and approved the submitted version.
